# Constipation and herbal medicine

**DOI:** 10.3389/fphar.2015.00073

**Published:** 2015-04-08

**Authors:** Norio Iizuka, Yoshihiko Hamamoto

**Affiliations:** ^1^Department of Kampo Medicine, Yamaguchi University Hospital, Ube, Japan; ^2^Department of Computer Science and Systems Engineering, Faculty of Engineering, Yamaguchi University, Ube, Japan

**Keywords:** constipation, herb, herbal medicine, Kampo, traditional medicine

## Abstract

Constipation is characterized by a variety of bowel symptoms such as difficulty passing stool, hard stool, and a feeling of incomplete evacuation. The multifactorial causes of constipation limit the clinical efficacy of current conventional treatments that use a single drug that acts through only one pathway. To complement the shortcomings of the current Western medical model and provide a complete holistic approach, herbal medicines capable of targeting multiple organs and cellular sites may be used. In Japan, many herbs and herbal combinations have traditionally been used as foods and medicines. Currently, Japanese physicians use standardized herbal combinations that provide consistent and essential quality and quantity. This review highlights representative Japanese herbal medicines (JHMs), Rhei rhizoma-based JHMs including Daiokanzoto and Mashiningan, and Kenchuto-based JHMs including Keishikashakuyakuto and Daikenchuto, which coordinate the motility of the alimentary tract. This review provides a framework to better understand the clinical and pharmacological efficacies of JHMs on constipation according to the unique theory of Japanese traditional medicine, known as Kampo medicine.

## Introduction

Constipation is characterized by a variety of bowel symptoms such as difficulty passing stool, hard stool, and feelings of incomplete evacuation. It is reported that 12–19% of Americans (Eoff, 2008) and 14% of Asians (Cheng et al., 2003) suffer from constipation, with symptoms varying by geographic location (Talley et al., 1993).

The multifactorial causes of constipation limit the clinical efficacy of current conventional Western treatments since these drugs act through a single pathway (Gallagher and O’Mahony, 2009). To complement these shortcomings and provide a complete holistic approach, herbal medicines capable of targeting multiple organ sites may be used (Suzuki et al., 2009). In Japan, many traditional herbs and herbal combinations are used as foods and medicines (Iizuka et al., 2003), and physicians currently use standardized herbal combinations with consistent quality and quantity of constituents (Yakubo et al., 2014).

This four-part review provides a framework to better understand the clinical and pharmacological efficacy of Japanese herbal medicines (JHMs) on constipation by: (1) explaining the unique theory of Japanese traditional medicine, or Kampo medicine (KM); (2) summarizing JHMs used for constipation; (3) explaining the clinical application and pharmacological action of Rhei Rhizoma-based JHMs; and (4) explaining the clinical application and pharmacology of Kenchuto-based JHMs.

## The Unique Theory of Japanese Traditional Medicine, or Kampo Medicine

Kampo medicine is another term for traditional Japanese medicine based on traditional Chinese medicine (TCM; Tang et al., 2008). The history of KM can be traced to about the fifth or sixth century, when TCM was likely introduced to Japan via Korea. At present, 148 government-regulated prescription JHMs are covered by Japanese national health insurance. These JHMs are high-quality herbs in combinations and proportions fixed and standardized according to classical TCM literature (Watanabe et al., 2011). Concomitant use of JHMs and various Western drugs is permitted in daily clinical practice under Japanese national health insurance.

There is a marked difference between Western medicine and traditional Asian medicines such as TCM, KM, and traditional Korean medicine. Western medicine specifically and efficiently attacks abnormal organs or cells by targeting the cause of the disease. More specifically, Western medicine focuses on pathogenesis rather than host reaction. In contrast, KM is concerned with the host’s reaction to the pathogen, and thus focuses on the host’s inherent ability to promote health by targeting multiple organs or cells concurrently. This is largely due to the fact that JHMs contain a combination of herbs, and thus a vast array of constituents. An evidence-based approach fails to assess efficacy when studying individualized medicines under the KM system because the end-points are somewhat unclear. This is an issue that requires further investigation in future studies.

Similar to TCM, KM uses patterns (*Sho* in Japanese, and *Zheng* in Chinese) to determine a suitable herbal combination for each patient. While TCM is based on the theory of the Ming Dynasty, KM was separated from this theory and then reestablished based on a different theory, Shang Han Lun, during the Edo period (Yakubo et al., 2014). While organ systems are very important for determining medication patterns in TCM, they are not utilized in KM because Japanese KM specialists wish to avoid overlap with terms used in Western medicine. Thus, KM patterns are quite unique. One possible reason for this may be the fact that TCM prescriptions are individualized at the herbal level, while KM prescriptions are individualized at the formula level (Yakubo et al., 2014).

For diagnosis in KM, several parameters are provided to formulate medication patterns, including yin-yang, deficiency-excess, cold-heat, interior-exterior, six stages of acute febrile diseases and Qi-blood-fluid (Sato et al., 2005; Yakubo et al., 2014). To assess and better understand patient status, KM defines chronic health conditions as, for example, deficiency, intermediate (i.e., between deficiency and excess), and excess patterns in the whole body and Qi-blood-fluid. Qi, or life energy, is sourced from food and air. There are three types of abnormal Qi patterns: Qi deficiency, Qi stagnation, and Qi counterflow. Blood is a red fluid moved by Qi. There are two types of abnormal blood patterns: blood deficiency and blood stasis. Fluid, in contrast to blood, is a colorless and transparent liquid. In KM, illness is caused by an imbalance of these three elements. Under KM theory, constipation is thought to be caused mainly by a deficiency pattern, specifically Qi deficiency, and other patterns (intermediate or excess) caused by Qi stagnation, blood deficiency, or blood stasis (Table 1). Physicians specializing in KM can individualize constipation treatments using the following four traditional examination methods: inspection, listening and smelling, interviewing, and palpation (Sato et al., 2005).

**TABLE 1:** **Japanese herbal medicines (JHMs) and Kampo patterns**.

Japanese herbal medicines (JHMs)	Class	Kampo patterns	Qi deficiency	Qi stagnation	Qi counterflow	Blood deficiency	Blood stasis	Fluid excess
Daiokanzoto	1	Intermediate						
Tokakujokito	1	Excess					•	
Bofutsushosan	1	Excess		•				
Tsudosan	1	Excess					•	
Mashiningan	1	Intermediate						
Junchoto	1	Intermediate				•		
Daikenchuto	2	Deficiency	•					
Keishikasyakuyakuto	2	Deficiency	•					
Shokenchuto	2	Deficiency	•					
Keishikasyakuyakudaioto	2	Deficiency	•					

Class 1: Rhei-Rhizoma-based JHMs; Class 2: Kenchuto-based JHMs.

## JHMs Used for Constipation

**FIGURE 1:**
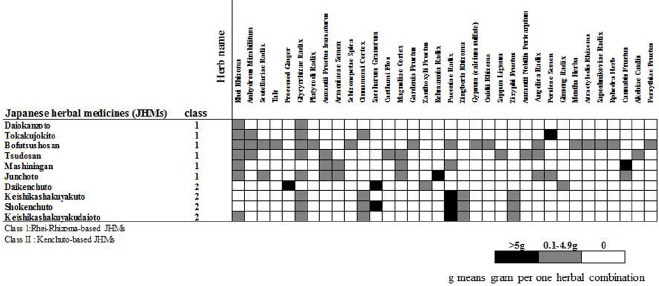
**Representative Japanese herbal medicines (JHMs) used for constipation.** Note that there are two representative JHMs, Rhei-Rhizoma-based JHMs (class 1) and Kenchuto-based JHMs (class 2) that are classified by combination patterns of herbs that are obtained from the package insert of Tsumura Kampo Formulation for Prescription (http://plaza.umin.ac.jp/∼kconsort/framepage.html).

**FIGURE 2:**
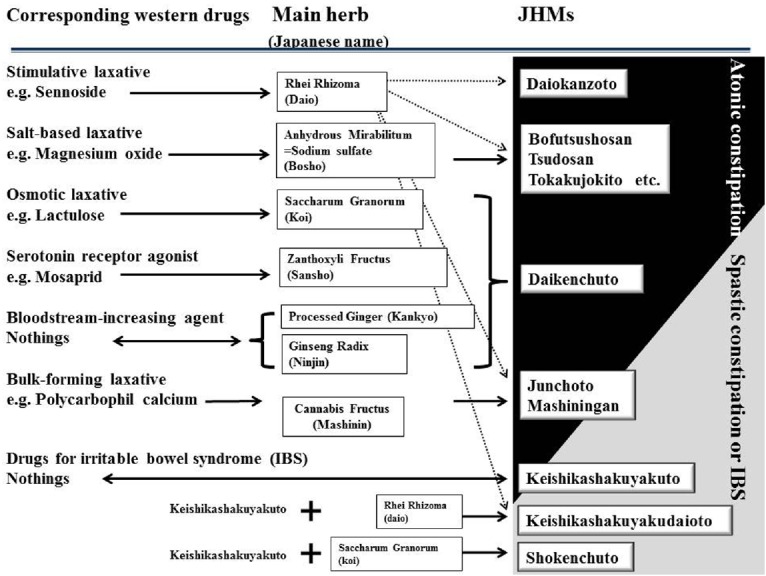
**Functional relation between Western drugs and Japanese herbal medicines (JHMs) used for constipation.** JHMs and their main herbs are listed along with the corresponding Western drugs. Notably, none of laxatives increase bloodstream in the alimentary tract (adapted from Iizuka, 2011).

Japanese herbal medicines are composed of various medicinal herbs. The pharmacological actions of JHMs in the body are largely determined by combinations and interactions between the herbs, which are used to treat KM patterns, including deficiency-excess and Qi-blood-fluid. Figure 1 shows representative JHMs and their ingredients (i.e., herbs) used for constipation. There are two classes of JHMs, Rhei Rhizoma-based JHMs (class 1) and Kenchuto-based JHMs (class 2), both of which are frequently used for constipation (Figure 1; Table 1). Figure 2 also depicts the functional pharmacological relationship between Western drugs and the primary herbs used in JHMs. Information concerning herbs in KM were obtained from an online list of package inserts (http://plaza.umin.ac.jp/∼kconsort/framepage.html).

Physicians specializing in KM usually use Rhei Rhizoma-based JHMs (class 1) to treat constipated patients with excess or intermediate patterns, most of whom show atonic constipation (Iizuka, 2011; Table 1; Figure 2). One possible reason for this is that sennoside A, aloe-emodin, and rhein, which are the main components of Rhei Rhizoma, have anti-inflammatory activities (He et al., 2013), and as a result, draw heat from the patient. Therefore, their pharmacological actions may cause harm in constipated patients with deficiency and cold patterns.

The word “Kenchuto” means an herbal combination that can improve the dysfunction of the alimentary tract (Sato et al., 2005). Kenchuto is classified into two types, Daikenchuto and Keishikashakuyakuto, based on herb combinations. Interestingly, although these two types of JHMs have different herb combinations, they have similar functions in terms of promoting bowel movements (Figure 1). KM physicians typically use Kenchuto-based JHMs (class 2) to treat constipated patients with deficiency and cold patterns who also report general fatigue due to Qi deficiency (Table 1). In Japan, Kenchuto-based JHMs are frequently prescribed when patients with deficiency and cold patterns develop constipation-predominant irritable bowel syndrome (IBS; Sato et al., 2005). In particular, contraction of the rectus abdominis muscle (*Fukuhikokyu* in Japanese) is often found in patients with constipation-predominant IBS. KM physicians recognize empirically that Paeoniae Radix, a main component of Kenchuto-based JHMs, promotes relaxation of the rectus abdominis muscle (Sato et al., 2005) as well as smooth muscle of the intestinal tract. Therefore, for patients with IBS, contractions of the rectus abdominis muscle can be used as a selection criterion for Kenchuto-based JHMs.

## Clinical Application and Pharmacology of Rhei-Rhizoma-Based JHMs

Rhei Rhizoma (Rhubarb in English and *Daio* in Japanese) is one of the most frequently used herbs for constipation throughout the world. Rhei Rhizoma contains dianthrone glucosides (sennosides A to F) and anthraquinones (e.g., rhein, aloe-emodin, emodin, physcion, chrysophanol; Hardcastle and Wilkins, 1970; Dreessen et al., 1981; Ngoc et al., 2008). Among these components, sennosides (i.e., stimulative laxatives), have been well documented for their pharmacological action on constipation (de Witte, 1993). Sennosides A and B play a central role in the motility of the alimentary tract as prodrugs that are converted to an active principle, rheinanthrone, by intestinal microbiota (Hardcastle and Wilkins, 1970; Dreessen et al., 1981). A recent study showed that sennoside A may exert a laxative effect by inhibiting water transfer from the intestinal tract to the vascular side via decreasing aquaporin-3 expression in the colon (Kon et al., 2014).

Daiokanzoto is a representative *Rhei rhizoma*-based JHM that comprises two herbs (*Rhei rhizoma* and *Glycyrrhizae radix*). It is widely used to treat constipated patients with intermediate patterns (Table 1). The pharmacological action of Daiokanzoto appears similar to that of sennoside, a main component of Rhei Rhizoma (Figure 2). Inversely, a patient’s response to sennoside may predict the clinical efficacy of Rhei Rhizoma-based JHMs. For example, KM physicians would not empirically prescribe Daiokanzoto for constipated patients deemed unresponsive to sennoside in the medical interview (Iizuka, 2011). Alternatively, Daiokanzoto has beneficial effects on oral mucositis, a disease that results from increased cell death induced by chemotherapeutic agent 5-fluorouracil (5-FU; Yoshida et al., 2014). Therefore, Daiokanzoto may improve both constipation and quality of life in cancer patients treated with 5-FU-based chemotherapy, although further studies are required to gain deeper insight into its pharmacological actions.

Bofutsushosan, Tsudosan, and Tokakujokito are composed of two main herbs: Rhei Rhizoma and Anhydrous Mirabilitum (*Bosho* in Japanese; Figures 1 and 2). Anhydrous Mirabilitum is a sodium sulfate similar to magnesium sulfate that acts as a salt-based laxative (Figure 2). Bofutsushosan, Tsudosan, and Tokakujokito are considered strong laxatives due to their stimulative and salt-based functions (Iizuka, 2011). Among these three JHMs, Tsudosan and Tokakujokito are used for constipated patients with blood stasis (*Oketsu* in Japanese; Table 1). Several herbs (Carthami Flos, Sappan Lignum, Angelica Radix, and Persicae Semen) used in Tsudosan and Tokakujokito improve blood stasis by inhibiting blood coagulation and causing vasodilation. For example, Angelica Radix contains coumarin derivatives, which have inhibitory effects on platelet aggregation and blood coagulation (Lee et al., 2003). Bofutsushosan is also used for constipated patients with Qi stagnation (*Kitai* in Japanese; Table 1). Daiokanzoto and these three JHMs are frequently used in patients with atonic constipation without deficiency and cold patterns.

Junchoto and Mashiningan are unique Rhei Rhizoma-based JHMs that contain small amounts of Rhei Rhizoma and Cannabis Fructus (*Mashinin* in Japanese; Figure 1). In Japan, KM physicians prescribe Junchoto and Mashiningan exclusively for elderly patients who have spastic constipation (Figure 2). Cannabis Fructus contains large amounts of fatty oils, including olein, linolein, and linolenin, with actions similar to the bulk-forming laxative, polycarbophil calcium (Montserrat-de la Paz et al., 2014; Figure 2). In most patients with constipation, Junchoto or Mashiningan soften stool. Generally, constipated stool is dry, hard, and difficult to pass. Therefore, the combination of Rhei Rhizoma, which promotes movement of the alimentary tract, and Cannabis Fructus, which softens stool, may prove effective for any type of constipation, including atonic and spastic constipation (Figure 2). Mashiningan has been proven efficacious for functional constipation in a randomized double-blind, placebo-controlled study (Cheng et al., 2011). In that study, 120 patients with functional constipation were randomized into two groups, a Mashiningan (Hemp Seed Pill) and a placebo group. Responder rates for the Mashiningan and placebo groups were 43.3 and 8.3% during treatment and 30.0 and 15.0% at 8-week follow-up, respectively (*P* < 0.05). This suggests that Mashiningan increases complete spontaneous bowel movement, relieves the severity of constipation and straining to evacuate, and effectively reduces the need for a laxative.

## Clinical Application and Pharmacology of Kenchuto-Based JHMs

**FIGURE 3:**
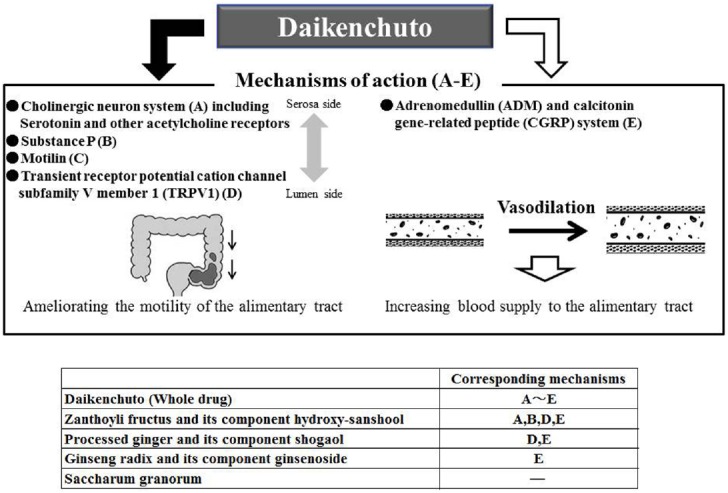
**Two major mechanisms of action of Daikenchuto.** Daikenchuto ameliorates the motility of the alimentary tract via four mechanisms **(A to D)** and increases blood supply to the alimentary tract with use of adrenomedullin (ADM) and calcitonin gene-related peptide (CGRP) system **(E)**. Notably, constituents playing an important role in individual mechanisms and system are identified.

Daikenchuto, one member of the Kenchuto-based JHM family, has a wide range of pharmacological actions and therefore is the most commonly prescribed JHM. It is composed of the following four herbs: processed ginger (*Kankyo* in Japanese), Ginseng Radix Rubra (*Ninjin* in Japanese), Zanthoxyli Fructus (Japanese pepper, *Sansho* in Japanese), and Saccharum Granorum (maltose powder derived from rice, *Koi* in Japanese; Kono et al., 2009; Figure 1). Due to a lack of relevant studies, the precise pharmacological action of Saccharum Granorum, a disaccharide with high osmotic pressure, remains unclear; however, it is not difficult to suspect that it might affect stool consistency and the motility of the alimentary tract in a manner similar to that of lactulose, an osmotic laxative (Figure 3).

The pharmacological actions of Daikenchuto, which are divided into two major putative mechanisms (Figure 3), have been well studied and documented (Shibata et al., 1999; Satoh et al., 2001a,b; Kono et al., 2008, 2009; Manabe et al., 2010). Daikenchuto increases blood supply to and improves the motility of the alimentary tract. The motility of the alimentary tract has been improved via the cholinergic neuron system (serotonin and nicotinic acetylcholine receptors), substance P, motilin, and transient receptor potential cation channel, subfamily V, member 1 (TRPV1) in several rodent and dog models with use of receptor antagonists or inhibitors (Nagano et al., 1999; Satoh et al., 2001a,b, 2003; Sato et al., 2004; Kikuchi et al., 2013). Several studies have shown that oral administration of Daikenchuto by healthy individuals increases plasma levels of substance P and motilin, which promote, either directly or indirectly, the motility of the alimentary tract (Nagano et al., 1999; Sato et al., 2004).

Zanthoxyli Fructus contains hydroxy-sanshools (alpha and beta), which act as a serotonin receptor agonist via the cholinergic neuron system to enhance intestinal peristalsis (Figure 3). As shown in Figure 2, its pharmacological action is similar to that of the serotonin receptor agonist mosapride. Indeed, it was shown that the increased intestinal motility induced by Daikenchuto was inhibited by several serotonin receptor antagonists (Shibata et al., 1999; Satoh et al., 2001a,b). A recent study by Kono et al. (2013) demonstrated that hydroxy-sanshools activate intestinal epithelial transient receptor potential cation channel, subfamily A, member 1 (TRPA1), which is highly expressed in enterochromaffin cells (serotonin-releasing cells), and may prove to be a novel target for constipation. In addition to the effects of these individual constituents, a recent study showed that Daikenchuto activates nicotinic acetylcholine receptors, which accounts for its effects on motility (Endo et al., 2014). Kikuchi et al. (2013) reported that pretreatment with atropine, hexamethonium, ondansetron (5-hydroxytryptamine-3 receptor antagonist), or capsazepine (TRPV1 antagonist), inhibited Daikenchuto-induced colonic contractions in a dog model. This suggests that orally administered Daikenchuto stimulates colonic motility via TRPV1, muscarinic, nicotinic, and 5-hydroxytryptamine-3 receptors. TRPV1, formerly known as vanilloid receptor 1, is known as the capsaicin receptor. Interestingly, 6-gingerol and 6-shogaol, which are extracted from processed ginger, have vanilloid structures, and possibly act as TRPV1 stimulators (Figure 3). It has been reported that hydroxy-sanshools extracted from Zanthoxyli Fructus also bind to TRPV1 (Koo et al., 2007). These constituents play an important role in promoting the movement of alimentary tract via sensory nerves.

Unlike other JHMs used for constipation, Daikenchuto also increases blood circulation (Figure 3). To the best of our knowledge, no other pharmaceutical laxatives produce this effect. Increased intestinal blood supply is very fascinating from the standpoint of long-term maintenance of digestive function. As shown in Figures 2 and 3, both 6-shogaol extracted from processed ginger and hydroxyl-alpha-sanshool extracted from Zanthoxyli Fructus, have been shown to increase intestinal circulation via calcitonin gene-related peptide (CGRP) and adrenomedullin (ADM; Kono et al., 2008). Ginseng Radix has also been shown to increase blood supply to other organs, including brain tissues (Kim et al., 2002). These findings help explain why KM physicians frequently use Daikenchuto to treat constipated patients with deficiency and cold patterns from poor blood supply.

Both gingerols and shogaols, constituents of processed ginger, have anti-inflammatory and circulatory effects in the alimentary tract via modulation of mitogen-activated protein kinase (MAPK), protein kinase B (Akt), and NF-κB activities (Hung et al., 2009; Wu et al., 2010; Huang et al., 2011; Li et al., 2013). Daikenchuto has been shown to significantly attenuate mucosal damage and adhesion in inflamed colon, and to inhibit elevated levels of proinflammatory cytokines TNFα and IFNγ in the colon in animal models of Crohn’s disease (Kono et al., 2010). This pharmacological action is explained in part by increased levels of ADM in small and large intestinal epithelial cells after Daikenchuto administration (Kono et al., 2010). These findings suggest that Daikenchuto has anti-colitis effects via the orchestration of several anti-inflammation pathways, which makes it suitable for treating patients with IBS or other inflammatory bowel diseases. The clinical application of these findings is currently underway in many hospitals throughout Japan, and the clinical efficacy of Daikenchuto will be elucidated in future studies.

Secondary members of the Kenchuto-based JHM family include Keishikashakuyakuto, Keishikashakuyakudaioto, and Shokenchuto, which are more frequently used in patients with constipation-predominant IBS (Sato et al., 2005; Oka et al., 2014). It is intriguing that Keishikashakuyakuto has shown antidiarrheal effects via the inhibition of excessively accelerated small intestinal movement (Saitoh et al., 1999); it has also been effective for relieving abdominal pain in patients with diarrhea-predominant IBS (Sasaki et al., 1998). Taken together, these reports suggest that Keishikashakuyakuto likely normalizes both accelerated and inhibited intestinal movements. This dual effect illustrates how certain herbal combinations, such as JHMs with multiple components, can help maintain host homeostasis.

Keishikashakuyakuto is composed of the following five herbs: Cinnamomi Cortex, Paeoniae Radix, Zingiberis Rhizoma, Zizyphi Fructus, and Glycyrrhizae Radix (Figure 1). Among these herbs, Paeoniae Radix and Glycyrrhizae Radix play a central role in ameliorating bowel dysfunction in patients with IBS. These two herbs has been shown to suppress the neurogenic contractions of the ileum induced by electrical stimulation and ganglionic-stimulating agents in guinea pigs via inhibition of acetylcholine (Ach) release from cholinergic nerves and inhibition of Ach action on ileum smooth muscle (Maeda et al., 1983). Likewise, their anti-spasmodic effect on the human colon has been confirmed (Ai et al., 2006).

In addition to its spasmolytic and smooth muscle-relaxing effects (Takagi and Harada, 1969b; Hayashi et al., 1990; Tsuji et al., 2012), Paeoniae Radix has been shown to have anti-inflammatory and analgesic effects, to inhibit gastric acid secretion and stress-induced ulceration (Takagi and Harada, 1969a), and to have sedative (Takagi and Harada, 1969a,b) and antidepressant-like effects in rodent models (Mao et al., 2012). Mao et al. (2012) suggested that Paeoniae Radix has antidepressant-like effects, which could be mediated in part by inhibiting monoamine oxidase activity, hypothalamic-pituitary-adrenal axis activation, oxidative stress, and upregulated brain-derived neurotrophic expression.

Glycyrrhizin, a main component of Glycyrrhizae Radix, and its metabolite, 18β-glycyrrhetinic acid, have been reported as likely responsible for ameliorating dysfunctional glutamate transport in astrocytes via the inhibition of protein kinase activity (Kawakami et al., 2010). It has also been reported that after administration of 18β-glycyrrhetinic acid, about 13% passes through the blood-brain barrier (Mao et al., 2012). These findings suggest that 18β-glycyrrhetinic acid in the brain can scavenge excess glutamate via a transporter, which might be related to the pathophysiology of bipolar disorder, major depressive disorder (Hashimoto et al., 2007), and schizophrenia (Schobel et al., 2013). Interestingly, brain-gut interactions have been suggested to play a central role in the pathogenesis of IBS (Fichna and Storr, 2012). Therefore, Keishikashakuyakuto orchestrates brain-gut interactions in IBS patients, Paeoniae Radix, in addition its direct effect on the alimentary tract, has both sedative and antidepressant-like effects, and Glycyrrhizae Radix induces upregulation of astrocyte glutamate transport. Thus, JHMs prove useful because they are capable of targeting multiple and/or distant organs such as the brain or alimentary tract concomitantly.

Shokenchuto is composed of the five herbs used in Keishi-kashakuyakuto and Saccharum Granorum (Figures 2 and 3). Keishikashakuyakudaioto is composed of the five herbs used in Keishikashakuyakuto, plus a small amount of Rhei Rhizoma (Figures 2 and 3). Considering that Saccharum Granorum and Rhei Rhizoma both enhance intestinal movement, it seems that Shokenchuto and Keishikashakuyakudaioto are stronger laxatives than Keishikashakuyakuto. However, the manner in which these three JHMs play important roles in controlling intestinal movement remains unclear. Further studies are needed to elucidate their specific pharmacological action on constipation.

## Conclusion

The authors have reviewed classic JHMs used to treat constipation and compared the pharmacological actions of KM with those of Western drugs. In a single-center, randomized, and double-blind study, Daikenchuto was found to have no significant effect on gastrointestinal and colonic transit, rectal compliance, anal sphincter pressure or bowel function in female patients with functional constipation (Iturrino et al., 2013). The effectiveness of KM allows physicians to provide individualized treatment to constipated patients and offer holistic JHMs capable of targeting multiple organ and cellular sites. However, there are limitations in the evaluation of the clinical efficacy of JHMs in a randomized clinical study setting because a placebo formulation that matches the texture, flavor, and other characteristics of the active drug is not always available. In addition, it is difficult to accurately evaluate herbal efficacy on individual patients with the current study model. A new strategy for evaluating various herbal combinations, including JHMs, and exploring pharmacological data may be needed in order to gain deeper insight into current herb-based therapies.

### Conflict of Interest Statement

The authors declare that the research is partially supported by grants from TSUMURA & CO.
